# Anti-Inflammatory Effect of Sulfated Polysaccharides Isolated from *Codium fragile* In Vitro in RAW 264.7 Macrophages and In Vivo in Zebrafish

**DOI:** 10.3390/md20060391

**Published:** 2022-06-13

**Authors:** Lei Wang, Jun-Geon Je, Caoxing Huang, Jae-Young Oh, Xiaoting Fu, Kaiqiang Wang, Ginnae Ahn, Jiachao Xu, Xin Gao, You-Jin Jeon

**Affiliations:** 1 College of Food Science and Engineering, Ocean University of China, Qingdao 266003, China; leiwang2021@ouc.edu.cn (L.W.); xiaotingfu@ouc.edu.cn (X.F.); wkq@ouc.edu.cn (K.W.); xujia@ouc.edu.cn (J.X.); xingao@ouc.edu.cn (X.G.); 2 Department of Marine Life Sciences, Jeju National University, Jeju 63243, Korea; wpwnsrjs@naver.com; 3 Co-Innovation Center for Efficient Processing and Utilization of Forest Products, College of Chemical Engineering, Nanjing Forestry University, Nanjing 210037, China; hcx@njfu.edu.cn; 4 Food Safety and Processing Research Division, National Institute of Fisheries Science, Busan 46083, Korea; ojy0724@naver.com; 5 Fujian Provincial Key Laboratory of Breeding Lateolabrax Japonicus, Fujian 355299, China; 6 Department of Marine Bio Food Science, Chonnam National University, Yeosu 59626, Korea; gnahn@chonnam.ac.kr; 7 Marine Science Institute, Jeju National University, Jeju 63333, Korea

**Keywords:** *Codium fragile*, sulfated polysaccharides, anti-inflammatory activity, RAW 264.7 cells, zebrafish

## Abstract

In this study, the anti-inflammatory activity of sulfated polysaccharides isolated from the green seaweed *Codium fragile* (CFCE-PS) was investigated in lipopolysaccharide (LPS)-stimulated RAW 264.7 macrophages and zebrafish. The results demonstrated that CFCE-PS significantly increased the viability of LPS-induced RAW 264.7 cells in a concentration-dependent manner. CFCE-PS remarkably and concentration-dependently reduced the levels of inflammatory molecules including prostaglandin E_2_, nitric oxide (NO), interleukin-1 beta, tumor necrosis factor-alpha, and interleukin-6 in LPS-stimulated RAW 264.7 cells. In addition, in vivo test results indicated that CFCE-PS effectively reduced reactive oxygen species, cell death, and NO levels in LPS-stimulated zebrafish. Thus, these results indicate that CFCE-PS possesses in vitro and in vivo anti-inflammatory activities and suggest it is a potential ingredient in the functional food and pharmaceutical industries.

## 1. Introduction

Inflammatory responses are immune responses that protect the organs from infection and tissue injury. Inflammatory responses are also associated with the pathogenesis of various diseases such as diabetes, cardiovascular diseases, obesity, arthritis, stroke, and cancer [1]. Inflammatory responses are associated with the release of inflammatory molecules such as histamine, prostaglandins, nitric oxide (NO), bradykinin, and pro-inflammatory cytokines [2]. The over-generation of these inflammatory molecules can cause uncontrolled inflammation, which leads to chronic inflammation and promotes the development of chronic inflammation-related diseases [3,4,5]. Thus, the inhibition of the production of inflammatory molecules is thought as a way to control the development of inflammation.

Seaweeds are rich in bioactive compounds. Seaweed-derived compounds possess several health benefits such as antiviral, anti-aging, antioxidant, anti-cancer, anti-obesity, anti-inflammatory, and anti-hypertensive effects [6,7]. Sulfated polysaccharides isolated from seaweeds possess strong anti-inflammatory activities [8,9]. Lipopolysaccharide (LPS), a component of the cell wall in Gram-negative bacteria, stimulates inflammatory responses. Thus, LPS-stimulated in vitro and in vivo models were used to investigate the anti-inflammatory activities of natural products. Sanjeewa et al. isolated a sulfated polysaccharide from the brown seaweed *Sargassum horneri*, which could inhibit lipopolysaccharide (LPS)-stimulated inflammation in RAW 264.7 cells and zebrafish [10]. Cui et al. purified a sulfated polysaccharide from *Gelidium pacificum* Okamura, which could suppress LPS-stimulated inflammation in human monocytic cells [8]. Rodrigues et al. investigated the anti-inflammation effect of the sulfated polysaccharide isolated from *Caulerpa cupressoides* (Cc-SP2). The results suggested that Cc-SP2 effectively suppressed acute inflammation in mice [9].

*Codium fragile* is a popular edible green seaweed. *C. fragile* contains various bioactive compounds such as fatty acids, carbohydrates, pigments, phenolic compounds, and proteins [11,12]. The anti-coagulation, antioxidant, anti-angiogenesis, anti-obesity, and immunoregulatory activities of *C. fragile* have been reported [13,14,15]. In previous studies, we evaluated the antioxidant activity of sulfated polysaccharides isolated from an enzymatic digest of *C. fragile* (CFCE-PS). The results demonstrated that CFCE-PS significantly suppressed hydrogen peroxide-induced oxidative damage in in vitro and in vivo models [16]. To further investigate the bioactivities of CFCE-PS, we evaluated the anti-inflammatory activity of CFCE-PS in LPS-stimulated RAW 264.7 cells and zebrafish.

## 2. Results and Discussion

### 2.1. CFCE-PS Suppressed Cytotoxicity and Inflammatory Molecules Production in LPS-Induced RAW 264.7 Cells

Sulfated polysaccharides isolated from seaweeds possess strong anti-inflammatory and immunostimulatory effects [17,18]. The anti-inflammatory activities of sulfated polysaccharides isolated from seaweeds, such as *Saccharina japonica*, *Sargassum horneri*, *Padina commersonii*, and *Chnoospora minima* have been investigated in our previous studies [19,20,21,22]. The results further confirm the potential of seaweed-derived sulfated polysaccharides in anti-inflammatory effects. Thus, in the present study, we evaluated the anti-inflammatory activity of CFCE-PS in in vitro and in vivo models.

**Figure 1:**
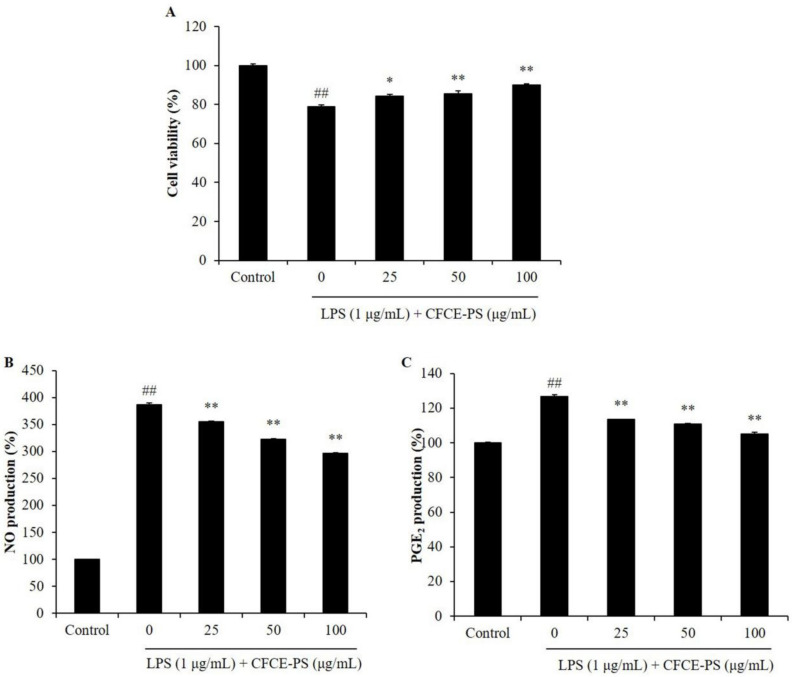
Effect of CFCE-PS on LPS-induced cytotoxicity in RAW 264.7 cells. (**A**) The viability of LPS-stimulated RAW 264.7 cells; the production levels of NO (**B**) and PGE_2_ (**C**) in LPS-stimulated RAW 264.7 cells. The cells non-treated with CFCE-PS and LPS are referred to as the control group. The experiments were conducted in triplicate and the data are expressed as mean ± SE. * *p* < 0.05, ** *p* < 0.01 as compared to the LPS-stimulated group and ^##^ *p* < 0.01 as compared to control group.

**Figure 2:**
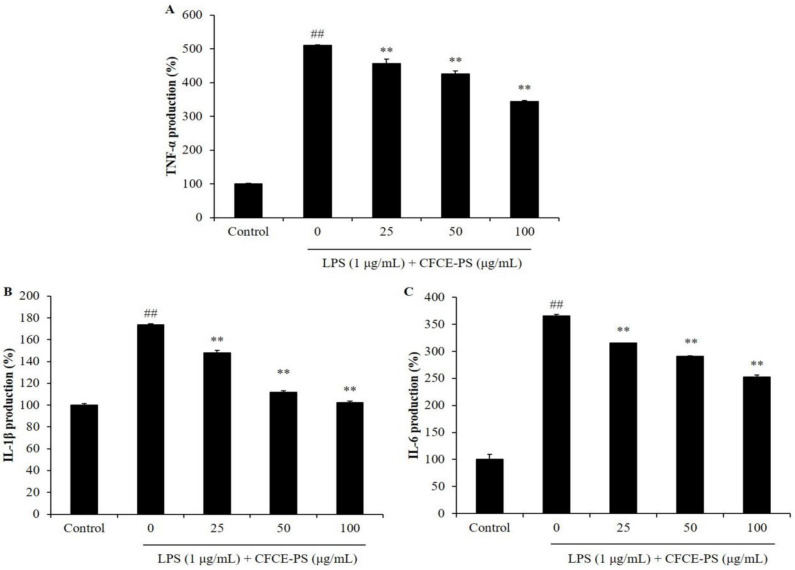
Effect of CFCE-PS on the production of pro-inflammatory cytokines in LPS-stimulated RAW 264.7 cells. (**A**) Production of TNF-α; (**B**) production of IL-1β; and (**C**) production of IL-6. The cells non-treated with CFCE-PS and LPS are referred to as the control group. The experiments were conducted in triplicate and the data are expressed as mean ± SE. ** *p* < 0.01 as compared to LPS-stimulated group and ^##^ *p* < 0.01 as compared to control group.

In the present study, the effects of CFCE-PS on LPS-induced cytotoxicity and inflammatory molecule production were evaluated. As shown in Figure 1A, the viability of LPS-stimulated RAW 264.7 cells was decreased to 78.84% compared to the cells non-treated with sample and LPS (control group, 100%), whereas the viabilities of the LPS-treated RAW 264.7 cells were increased to 84.36, 85.55, and 89.90% by CFCE-PS at concentrations of 25, 50, and 100 μg/mL, respectively (Figure 1A). In addition, LPS significantly stimulated the production of NO and prostaglandin E_2_ (PGE_2_) in RAW 264.7 cells (Figure 1B,C). However, the production of NO and PGE_2_ in LPS-treated RAW 264.7 cells was significantly reduced by CFCE-PS treatment in a concentration-dependent manner (Figure 1B,C). As shown in Figure 2, LPS significantly stimulated the production of pro-inflammatory cytokines including interleukin-1 beta (IL-1β), tumor necrosis factor-alpha (TNF-α), and interleukin-6 (IL-6) in RAW 264.7 cells. However, the production of these pro-inflammatory cytokines in RAW 264.7 cells was effectively suppressed by CFCE-PS treatment in a concentration-dependent manner (Figure 2). These results indicate that CFCE-PS protected RAW 264.7 cells against LPS-stimulated cell death by inhibiting inflammatory molecule production.

Anti-inflammatory effects of the algal sulfated polysaccharides were related to their sulfated content and proportion of monosaccharides. Previous reports suggested that the polysaccharides contain high amounts of the sulfate group, fucose, and galactose, which could inhibit the LPS-induced inflammatory response in RAW 264.7 cells [23,24,25,26,27]. CFCE-PS contains 21.06% sulfate and 70.19% galactose, and significantly inhibited the production of the inflammatory molecules in LPS-stimulated RAW 264.7 cells. According to the previous and present results, CFCE-PS suppressed LPS-stimulated cytotoxicity and the production of inflammatory molecules in RAW 264.7 cells. This action may be due to it containing a high amount of the sulfate group and galactose.

### 2.2. Protective Effect of CFCE-PS against LPS-Stimulated Inflammatory Response in Zebrafish

**Figure 3:**
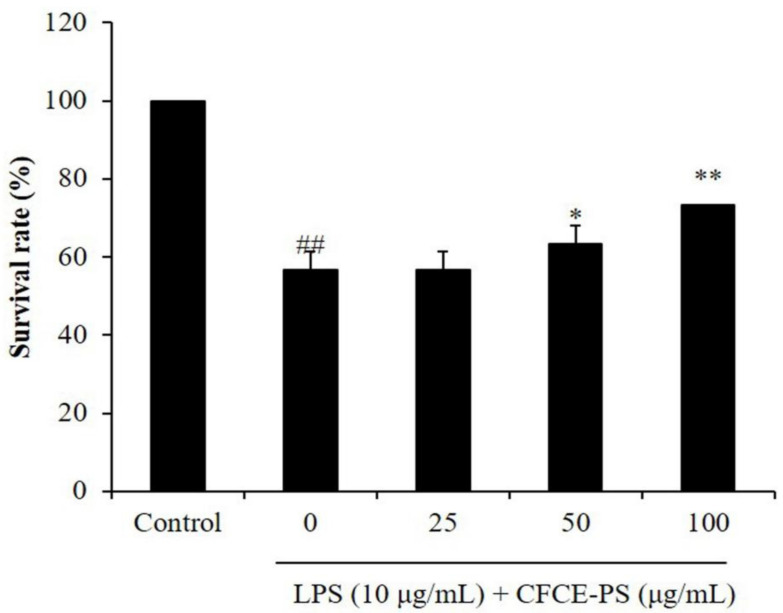
Survival rate of zebrafish after being treated with CFCE-PS or/and with LPS. The zebrafish non-treated with CFCE-PS and LPS are referred to as the control group. The experiments were conducted in triplicate and the data are expressed as the mean ± SE. * *p* < 0.05, ** *p* < 0.01 as compared to the LPS-stimulated group and ^##^ *p* < 0.01 as compared to the control group.

**Figure 4:**
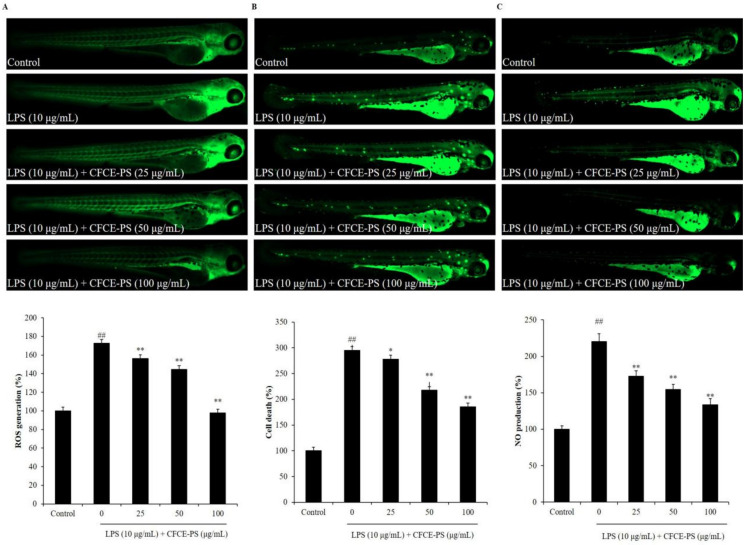
Effect of CFCE-PS on inflammatory responses in LPS-induced zebrafish. (**A**) ROS level of LPS-stimulated zebrafish; (**B**) cell death of LPS-stimulated zebrafish; and (**C**) NO production in LPS-stimulated zebrafish. The zebrafish non-treated with CFCE-PS and LPS are referred to as the control group. The relative amounts of ROS, cell death, and NO of zebrafish were measured using ImageJ software. The experiments were conducted in triplicate and the data are expressed as the mean ± SE. * *p* < 0.05, ** *p* < 0.01 as compared to the LPS-treated group and ^##^ *p* < 0.01 as compared to control group.

LPS-stimulated zebrafish embryo has been successfully used to investigate the in vivo anti-inflammatory effects of sulfated polysaccharides in our previous studies [21]. Therefore, in this study, the LPS-stimulated zebrafish embryo was used as the in vivo model to investigate the anti-inflammatory effect of CFCE-PS. As shown in Figure 3, the survival rate of LPS-stimulated zebrafish decreased to 56.67% compared to the control group (100%), whereas the survival rates of zebrafish treated with 50 and 100 μg/mL CFCE-PS were remarkably increased to 63.33 and 73.33%, respectively (Figure 3). In addition, the ROS level of LPS-treated zebrafish was increased to 172.65% compared to the control group (100%). However, the levels of ROS in CFCE-PS-treated zebrafish were significantly decreased in a dose-dependent manner (Figure 4A). As shown in Figure 4B, LPS significantly stimulated cell death in zebrafish, whereas the cell death levels of zebrafish treated with 25, 50, and 100 μg/mL CFCE-PS were decreased from 295.22% to 277.72, 217.68, and 185.58%, respectively (Figure 4B). Furthermore, the NO production level of zebrafish stimulated with LPS was increased to 220.45% compared to the control group (100%). However, the NO level of LPS-stimulated zebrafish was reduced to 172.99, 154.53, and 133.51% by the treatment of 25, 50, and 100 μg/mL CFCE-PS, respectively (Figure 4C). These results demonstrate that CFCE-PS remarkably protected zebrafish against LPS-stimulated inflammation.

In summary, in this study, the in vitro and in vivo anti-inflammatory effects of CFCE-PS were evaluated in LPS-stimulated RAW 264.7 cells and zebrafish. The results indicate that CFCE-PS inhibited LPS-induced inflammatory response by inhibiting the production of NO and PGE_2_, and decreasing the levels of pro-inflammatory cytokines in RAW 264.7 cells. In addition, the in vivo test showed that CFCE-PS remarkably suppressed the survival rate and reduced the levels of ROS, cell death, and NO in LPS-stimulated zebrafish. These results indicate that CFCE-PS possesses in vitro and in vivo anti-inflammatory effects. It could be used as a functional material in food and pharmaceutical industries.

## 3. Materials and Methods

### 3.1. Reagents and Chemicals

LPS, MTT, and DMSO were purchased from Sigma-Aldrich (St. Louis, MO, USA). Enzyme-linked immunosorbent assay (ELISA) kits used for measurement of IL-1β, TNF-α, PGE_2_, and IL-6 were purchased from R&D Systems Inc. (Minneapolis, MN, USA).

### 3.2. Preparation of CFCE-PS

*C. fragile* was collected in June 2019 from the coastal area of Jeju Island, South Korea. CFCE-PS was prepared based on the protocol described in the previous study [16]. CFCE-PS contained 76.84% sulfated polysaccharides, including 21.06% sulfate and 55.78% carbohydrates, which were composed of galactose (70.19%), arabinose (18.71%), glucose (9.10%), and xylose (1.99%).

### 3.3. Measurement of NO Level and Cell Viability

RAW 264.7 macrophages (RAW 264.7 cells, TIB-71^TM^) were purchased from ATCC (Manassas, WV, USA). Cells were seeded in a 24-well plate (1 × 10^5^ cells/mL) for 24 h. The cells were treated with 25, 50, and 100 µg/mL CFCE-PS. The cells non-treated with CFCE-PS and LPS are referred to as the control group. CFCE-PS-treated cells were incubated for 1 h and the plate was replaced with the media containing 1 µg/mL LPS. After 24 h incubation, the viability of LPS-treated RAW264.7 cells was measured using an MTT assay, and the production of NO was determined using a Griess assay [28].

### 3.4. ELISA

RAW 264.7 cells were seeded in a 24-well plate (1 × 10^5^ cells/mL) for 24 h. The cells were treated with 25, 50, and 100 µg/mL CFCE-PS. CFCE-PS-treated cells were incubated for 1 h and stimulated with 1 µg/mL LPS. After 24 h, the cell culture media was collected. The levels of pro-inflammatory cytokines and PGE_2_ were evaluated using ELISA kits [28].

### 3.5. Application of CFCE-PS and LPS to Zebrafish

The adult zebrafish were purchased from a commercial market (Seoul Aquarium, Korea) and maintained according to the condition described in our previous study [29]. After approximately 7–9 h of post-fertilization (hpf), the embryos were treated with 25, 50, and 100 μg/mL CFCE-PS. After 1 h of incubation, the embryos were incubated with the media containing 10 µg/mL LPS until 24 hpf. The zebrafish non-treated with CFCE-PS and LPS are referred to as the control group. The survival rate of zebrafish was measured at 3 days post-fertilization. The levels of cell death, ROS, and NO in LPS-induced zebrafish were measured in live zebrafish larvae by acridine orange, DCFH2-DA, and DAF-FM-DA staining, respectively [30].

### 3.6. Statistical Analysis

The experiments were conducted in triplicate and the data are expressed as the mean ± standard error (SE). One-way analysis of variance used to compare the mean values of each treatment using SPSS 20.0. Significant differences between the means were estimated using Tukey’s test.

## 4. Conclusions

In this study, the in vitro and in vivo anti-inflammatory activities of sulfated polysaccharides of the edible seaweed *C. fragile* (CFCE-PS) were investigated. The results demonstrated that CFCE-PS effectively inhibited LPS-stimulated inflammation in vitro in RAW 264.7 cells and in vivo in zebrafish. These results suggest that CFCE-PS is a potential anti-inflammatory ingredient in functional food and the pharmaceutical industry. However, to develop CFCE-PS as a therapeutic agent to treat inflammatory-related diseases, the clinical study is vital in further research.

## Data Availability

Not applicable.
